# Invasive solid papillary carcinoma with neuroendocrine differentiation of the breast: a case report and literature review

**DOI:** 10.1186/s40792-020-00905-x

**Published:** 2020-06-19

**Authors:** Xue Lin, Yoshiaki Matsumoto, Tomomi Nakakimura, Kazuo Ono, Shigeaki Umeoka, Masae Torii, Hiroshi Yoshibayashi, Masakazu Toi

**Affiliations:** 1 Department of Breast Surgery, Japanese Red Cross Wakayama Medical Center, 4-20 Komatsubara-dori, Wakayama, 640-8558 Japan; 2 Department of Breast Surgery, Kyoto University Hospital, 54 Kawaharacho, Syogoin, Sakyo-ku, Kyoto, 606-8507 Japan; 3 Department of Diagnostic Pathology, Japanese Red Cross Wakayama Medical Center, 4-20 Komatsubara-dori, Wakayama, 640-8558 Japan; 4 Department of Diagnostic Radiology, Japanese Red Cross Wakayama Medical Center, 4-20 Komatsubara-dori, Wakayama, 640-8558 Japan

**Keywords:** Invasive solid papillary carcinoma, Neuroendocrine differentiation, Breast, Metastasis

## Abstract

**Background:**

Solid papillary carcinoma (SPC) of the breast is a rare breast cancer that accounts for less than 1% of all breast cancers. The optimal clinical management of SPC remains controversial. Here, we report a case of invasive SPC with neuroendocrine differentiation in addition to review of the current literature.

**Case presentation:**

A premenopausal 46-year-old female presented with a mass in her left breast that tended to increase in size over a 10-month period. Mammography and ultrasonography revealed a mass in the left upper-inner quadrant. The resulting images suggested a category 3 breast tumor according to the Breast Imaging Reporting and Data System (BI-RADS). A core needle biopsy (CNB) was performed, and the pathological findings showed a solid papillary pattern and atypical cells suggestive of noninvasive SPC. After a left partial mastectomy and sentinel lymph node biopsy (SLNB), the specimens were sent for histopathological analysis for further investigation. Postoperative pathological findings suggested invasive SPC. Whole-breast radiation therapy and adjuvant hormonal therapy were performed as postoperative treatments. Three years after surgery, multiple lung metastases were detected, and the patient was treated with a gonadotropin-releasing hormone agonist and an aromatase inhibitor. Five months later, multiple liver metastases and bone metastases appeared, and oral 5-fluorouracil was chosen for the subsequent treatment. The patient has been treated for 5 years to date, and she is continuing to take oral 5-fluorouracil and is alive without any further disease progression.

**Conclusions:**

We report a rare case of premenopausal invasive SPC with multiple metastases. Further study is needed to clarify the molecular characteristics and clinical behaviors of SPC and to explore the optimal treatment strategy.

## Background

Solid papillary carcinoma (SPC) is a rare mammary papillary lesion that is difficult to pathologically diagnose [1, 2] and was first reported in 1995 by Maluf and Koerner [3]. SPC is a low-grade breast tumor that originates from expanded ducts and comprises morphologically well-circumscribed solid nodules separated by fibrovascular cores [1, 2, 4]. SPC is a rare disease that mainly affects postmenopausal women; indeed, it is relatively rare for a woman to be affected before the age of 50, and the average age of diagnosis with SPC is approximately 70 years old [1–6]. Although SPC is considered to be carcinoma in situ by the latest WHO classification, some studies have suggested that SPC is actually an invasive cancer because of the absence of myoepithelial cells at the tumor periphery [2, 7, 8]. SPC accounts for approximately 1% of all breast cancers. At the time of diagnosis, approximately 90% of cases are localized lesions, 8% involve lymph node metastases, and less than 0.4% present with distant metastases [2, 8, 9]. In this article, we report a case of a 46-year-old patient with invasive SPC who developed multiple distant metastases 3 years after surgery.

## Case presentation

A 46-year-old woman received a mammogram, which showed a high-density mass less than 10 mm in her left breast. Subsequent breast ultrasonography (US) showed a well-demarcated and low-echogenic mass that measured 8 mm in the left upper-inner quadrant.

**Fig. 1:**
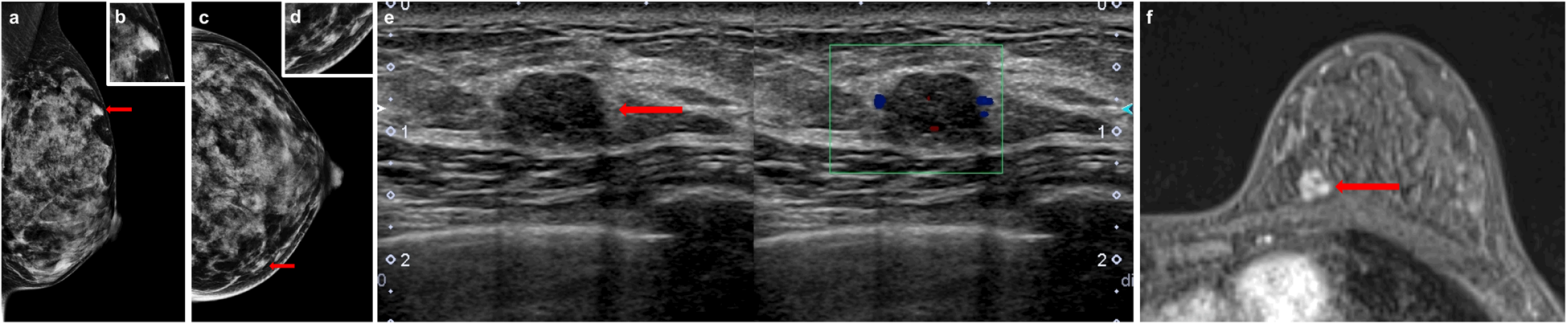
Imaging findings. Mammogram showing a round, high-density mass in the left MLO-M area (**a**) and CC-I area (**c**). **b**, **d** Enlargements of the areas are indicated by red arrows. US shows a regular, circumscribed, hypoechoic, homogenous lesion with abundant blood flow in the A area of the left breast (**e**). Dynamic MRI demonstrates an ill-demarcated mass that measures 8 mm × 7 mm in the A area of the left breast (**f**) without daughter lesions or enlarged lymph nodes

**Fig. 2:**
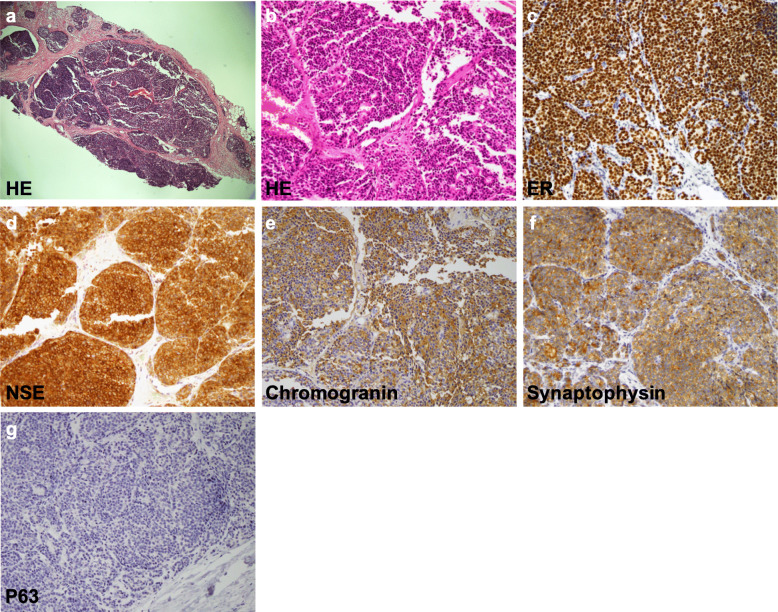
Core needle biopsy histological findings of the tumor (**a**) and (**b**) HE-stained sections at × 4 and × 20 magnification. **a** Low-power view illustrates a solid papillary carcinoma. **b** High-power view demonstrates the rosette structure of a solid papillary growth pattern and a network of thick fibrovascular cores among proliferating atypical malignant cells. **c** Positive immunohistochemical staining findings for the estrogen receptor. Positive immunohistochemical staining findings for the following markers demonstrate the neuroendocrine component of the tumor: **d** neuron-specific enolase, **e** chromogranin, and **f** synaptophysin. **g** Negative immunohistochemical staining for the myoepithelial marker p63 (**a**: × 4, **b**–**g**: × 20)

The results of the initial mammography and repeated US at her family clinic revealed that the mass had increase in size at a 10-month follow-up, and the patient was subsequently referred to our hospital. Mammography (Fig. 1a–d) and US (Fig. 1e) were repeated, and the US findings confirmed an increased mass size with abundant blood flow suggestive of a malignant tumor. Contrast-enhanced MRI demonstrated an ill-demarcated mass that measured 8 mm × 7 mm in the A area of the left breast (Fig. 1f). There were no apparent daughter lesions, intraductal findings, or lymph node swelling. The core needle biopsy (CNB) pathology showed clusters of atypical cells proliferating in solid and papillary patterns with a high nucleocytoplasmic (N/C) ratio (Fig. 2a, b). In addition, immunohistochemistry was performed with various monoclonal antibodies to confirm the diagnosis. The tumor was ER positive (Fig. 2c) and PgR negative. Regarding neuroendocrine markers, the SPC cells were positive for neuron-specific enolase (NSE), chromogranin, and synaptophysin (Fig. 2d–f), and negative for CD56 and S-100p antibodies. There were no myoepithelial cells on the outer edge of the tumor; this was confirmed by negative immunohistochemical (IHC) staining for myoepithelial markers, which included CD10, p63 (Fig. 2g), and α-smooth muscle actin. No invasive carcinoma was found. Considering the imaging and histology findings together, the preoperative diagnosis of the tumor was neuroendocrine SPC, and its stage was cTiscN0M0.

**Fig. 3:**
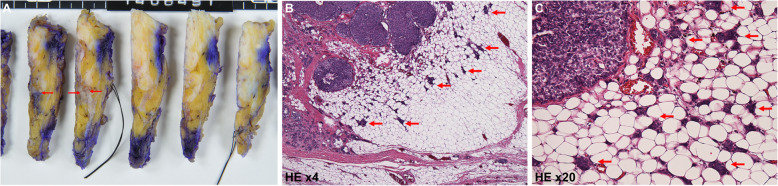
Postoperative histological findings of the tumor. **a** Postoperative surgical specimen of the left breast tissue with a visible tumor (red arrow). **b** HE-stained section of the tumor at × 4 magnification. **c** HE-stained section of the tumor at × 20 magnification. The HE-stained sections illustrate a clear infiltration that suggests an invasive solid papillary carcinoma. The invasive components that spread out from the solid structure are visible (red arrow, **b**, **c**)

The patient underwent a partial mastectomy of the left breast with sentinel lymph node biopsy (SLNB). For further confirmation, the specimens were sent for histopathological analysis (Fig. 3a), which showed well-circumscribed solitary nodules with neuroendocrine components. Microscopic observation revealed dilated ducts filled with a high proportion of proliferating cells that contained a necrotic vascular stroma. Some tube-like structures were also observed, and these findings corresponded with SPC. The HE-stained sections suggested an obvious infiltration pattern, with a distribution that spread out from the lobular unit. Invasion was observed as irregularly shaped tumor nests that invaded the surrounding stroma (Fig. 3b, c). The tumor was 8 mm × 7 mm in size, and the final diagnosis was SPC with invasion and massive SPC in situ.

In the immunohistochemical analysis, tumor cells were positive for NSE, chromogranin, and synaptophysin, which suggested that the tumor had a neuroendocrine feature. The myoepithelial markers CD10, P63, and α-smooth muscle actin were negative. Postoperative pathological findings suggested SPC with invasion. The tumor had an ER-positive, PgR-negative, and HER2-negative biological phenotype, while the Ki-67 labeling index was approximately 30%. The tumor exhibited an intermediate nuclear grade (grade II: 3-2-2) in the invasive components, and the stage was pT1b(8 mm)pN0(0/2, sn)M0, pStage I.

**Fig. 4:**
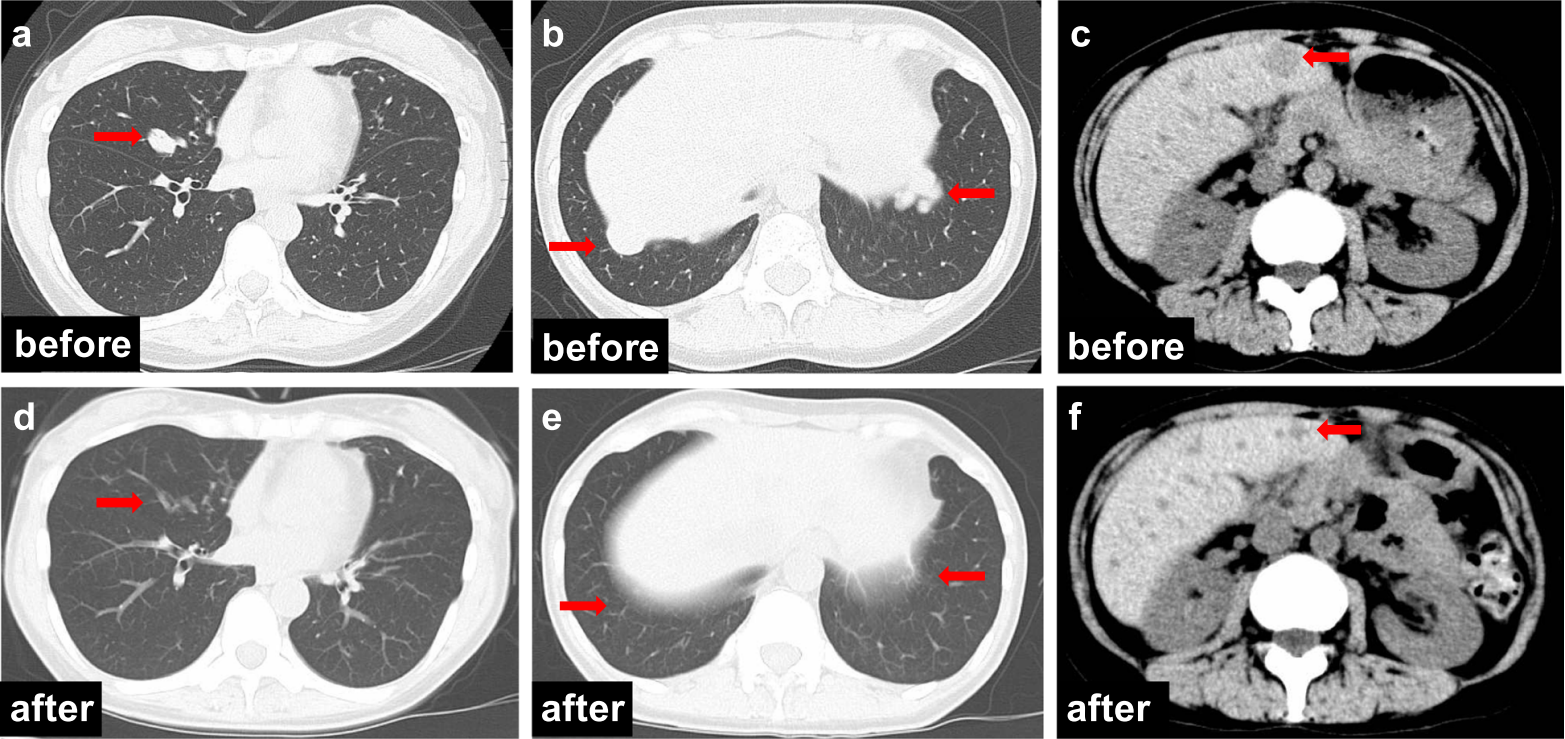
Whole-body CT before and after chemotherapy. Lung metastatic nodules (**a**, **b**) and liver tumors (**c**) were detected. According to treatment with oral 5-FU, the lung metastatic tumors completely disappeared (**d**, **e**), and the liver metastases remarkably regressed (**f**)

The postoperative treatment plan was determined based on the pathological diagnosis. Whole-breast radiation therapy followed by hormonal therapy (tamoxifen and a gonadotropin-releasing hormone (GnRH) agonist) was performed as adjuvant therapy. Three years after surgery, the patient developed a persistent cough with the absence of an inflammatory response, and a PET-CT examination showed multiple lung metastases. Systemic treatment was changed to a combination of an aromatase inhibitor and a GnRH agonist. Nevertheless, the metastatic tumors were aggressive, sizes of pulmonary lesions increased, and new liver and bone metastases appeared in the whole-body CT images 5 months after starting the treatment (Fig. 4a–c). We talked with the patient carefully about the merits and drawbacks of other treatments including chemotherapy, and the patient decided to undergo oral 5-FU treatment as the first-line chemotherapy. One year after the start of oral 5-FU treatment (S-1: 100 mg/body/day, 2 weeks on 1 week off), the lung metastases disappeared, and the sizes of liver metastases were significantly reduced in size (Fig. 4d–f). At present, the patient has taken the same dose of oral 5-FU treatment continuously for 2.5 years. No serious adverse events have been observed, and her quality of life (QOL) during the treatment currently appears to be favorable, more than 5 years after surgery.

## Discussion

SPC is an uncommon breast tumor that is usually detected in postmenopausal women between 60 and 80 years of age [2, 4, 9, 10]. In the described case, the patient was a 46-year-old premenopausal woman. In previously published literature, only 5.8% (9/156) of patients were premenopausal when SPC was diagnosed (Table 1); thus, the current SPC case is considered to be relatively unusual.

**Table 1:** Previous reports of SPC

Reference	Number of cases	% premenopausal (age^a^)	% of cases that showed distant metastasis
Maluf and Koerner [3]	20	0	5%
Otsuki et al. [4]	20	15% (31, 45, 48)	0
Nassar et al. [6]	58	1.7% (30)	8.6%
Nicolas et al. [11]	11	9.1% (48)	0
Foschini et al. [12]	13	7.7% (48)	n.a.
Eusebi et al. [13]	5	0	n.a.
Tosi et al. [14]	4	50% (45, 47)	n.a.
Cameselle et al. [15]	1	0	n.a.
Chang et al. [16]	1	0	n.a.
Masood et al. [17]	1	0	n.a.
Colella et al. [18]	1	0	n.a.
Chiang et al. [19]	13	0	n.a.
Leena et al. [20]	2	50% (44)	n.a.
Zhang et al. [21]	4	0	n.a.
Senel et al. [22]	1	0	n.a.
Domoto et al. [23]	1	0	n.a.
Rakha et al. [ 8]	30	n.a.	0
Tsang and Chan [24]	34	n.a.	0
Wei et al. [25]	21	n.a.	0
Zheng et al. [26]	32	n.a.	3.2%
Zhong et al. [27]	22	n.a.	0
**Total**	**295**	**5.8%**^**b**^**(9/156)**	**2.8%**^**b**^**(7/248)**

*n.a* not available

^a^Premenopausal patients’ age

^b^The percentage data was calculated with the exception of n.a. cases

SPC originates from the ductal epithelium, and previously, studies have reported that the SPC tumor size ranges from less than 10 to 150 mm [1–3, 6, 24]. Macroscopically, the tumors can be solitary or multiple and are well-circumscribed, nodular, and soft masses with hemorrhagic and cystic components. A gelatinous appearance may be grossly appreciated in tumors with mucinous differentiation [2, 4, 9]. Microscopically, SPC appears as proliferative nodules, each representing a duct filled with neoplastic proliferative components [2]. The components can be either ovoid or spindle-shaped and are rarely stream-like in appearance, similar to ductal hyperplasia [2, 6]. The tumor components grow in a solid pattern with an intermingled fibrovascular network [2, 3]. In addition, nuclear palisading and pseudorosette formation around capillary vessels are standard features [2–4, 6].

Approximately 50% of cases are associated with invasive carcinoma [2]. In previous studies, invasive components have been reported in 27.2–75% of cases [4, 6, 9, 28]. The invasion can be multifocal and may have a pattern that is neuroendocrine-like, pure or mixed colloid, tubular, or invasive ductal but is rarely lobular [2, 6, 28]. SPC may also express one of the myoepithelial markers, including P63, α-smooth muscle actin, and CD10, and the expression of the proliferative tumor marker Ki-67 is usually low (less than 10%) [6, 10, 28]. Most SPCs show features of a low or intermediate nuclear grade, and a high nuclear grade is only reported in a few cases [6, 28–30].

In the diagnosis of SPC, classification as in situ or invasive lesions is recommended [28]. Different studies have shown that SPC lacks myoepithelial cells at the tumor nodule periphery [2, 6, 9, 11, 24, 29]. In some studies, pathologists have routinely used the lack of peripheral myoepithelial cells to ascertain the invasion of papillary tumors, but this criterion is still controversial [2, 6]. In general, the invasive carcinoma component is diagnosed when malignant cells are clearly present beyond the solid tumor nodules [2, 31].

In the present case, the tumor size was determined microscopically and was 8 mm × 7 mm, and the tumor showed a solid, smooth margin against stromal tissue and a complete lack of staining of the myoepithelial markers at the periphery of the duct. Invasion was observed as irregularly shaped tumor nests that invaded the surrounding stroma, while the malignant cells were clearly spread out from the lobular unit of the adjacent SPC.

SPCs usually express a luminal phenotype [4, 6, 9, 29, 30] and are positive for ER and PgR but are negative for HER2 [2, 6]. The proliferation index is usually low, while SPCs are usually positive for neuroendocrine markers, including NSE, synaptophysin, and chromogranin, and negative for S-100p and CD56 [2, 3, 6]. Neuroendocrine differentiation has been reported in approximately half of all SPC cases in the current literature [4, 6, 9, 28–30]. It has been suggested that neuroendocrine differentiation might be a useful marker for the diagnosis of SPC [24, 29, 30]. However, not all SPC tumor cells exhibit neuroendocrine differentiation, and approximately 20% of invasive carcinomas of no special type also exhibit neuroendocrine differentiation. Therefore, neuroendocrine differentiation is clearly not necessary for the diagnosis of SPC [4, 29]. In this case, in concordance with the published literature, the tumor was positive for ER receptor, negative for PgR receptor, and negative for HER2. Additionally, NSE, chromogranin, and synaptophysin were positive, but S-100p and CD56 were negative. Neuroendocrine differentiation should be assessed carefully in the diagnosis of invasive SPC, the clinical implication of which remains unknown. Bogina et al. [32] and Kwon et al. [33] investigated neuroendocrine differentiation in breast carcinoma (NDBC) compared to non-NDBC. The results revealed that patients with NDBC showed worse disease-free survival and overall survival compared to non-NDBC. Several researchers also highlighted the poor prognostic outcome of invasive NDBC, although the association remains uncertain [28, 30, 34].

There are currently no evidence-based guidelines for the treatment of SPC, particularly for invasive disease, and as a result, the management of SPC is still controversial [8–10]. In invasive SPC, the prognosis may depend on the biological tumor features of the invasive component. Distant metastasis can be found without infiltration in the axillary lymph nodes [2, 6, 8]. Some authors have suggested that invasive SPC tumors are morphologically similar to SPC, but there is currently no clear evidence to ascertain whether the metastasis originates from the invasive SPC [1]. The management of SPC varies from breast-conserving surgery to mastectomy, while the role of SLNB is unclear. Axillary lymph node metastasis is thought to be present in approximately 3–5% of patients [1, 10]. Retrospective analysis of previous studies on SPC showed that the frequency of distant metastases was approximately 2.8% (7/248) (Table 1).

In the present case, breast-conserving surgery, radiotherapy, and endocrine therapy were performed. Although there was no axillary lymph node involvement, this patient recurred with during adjuvant endocrine treatment, suggesting that the tumor would be hormone resistant. According to the anatomical stage and pathological findings, this patient was assessed as being at a low-intermediate risk of recurrence, but she developed metastases early. The mechanism of SPC metastasis is largely unknown [28], but as a speculation, luminal B-like features, PgR negativity, neuroendocrine differentiation, and a relatively young patient age may be associated [28, 30, 32–35]. As described, neuroendocrine differentiation shows a higher propensity for recurrence and poor overall survival [32–34].

Oral treatment with 5-FU (S-1) was effective for this case. S-1 is well-tolerated, and its clinical usefulness has been shown in multiple trials [36–39]. Treatment with S-1 was feasible, and the QOL of this patient has been maintained for more than 2 years. Although these metronomic treatments might be efficient for invasive SPC, obviously, further studies are needed.

## Conclusions

In conclusion, we reported a case of invasive neuroendocrine-differentiated SPC of the breast that developed in a premenopausal woman. Recurrence with multiple metastases occurred during adjuvant endocrine therapy, following which, an oral 5-FU was effective against the metastases. Invasive SPC remains rare, and its biological and clinical behaviors are largely unknown. Therefore, further investigations, with molecular analysis and accumulation of clinical experience, are warranted.

## Data Availability

Not applicable.

## Abbreviations

SPC: Solid papillary carcinoma
N/C ratio: Nucleocytoplasmic ratio
SLNB: Sentinel lymph node biopsy
CNB: Core needle biopsy
ER: Estrogen receptor
PgR: Progesterone receptor
HER2: Human epidermal growth factor receptor 2
US: Ultrasonography
DCIS: Ductal carcinoma in situ
MRM: Modified radical mastectomy
NDBC: Neuroendocrine differentiation breast carcinoma
QOL: Quality of life
GnRH: Gonadotropin releasing hormone
5-FU: 5-Fluorouracil
NSE: Neuron-specific enolase
